# Clinical and pathogenic characteristics of infectious endophthalmitis in children: A retrospective analysis

**DOI:** 10.1097/MD.0000000000038456

**Published:** 2024-06-14

**Authors:** Peipei Jia, Jiayin Qin, Xiaolu Cao, Yixiang Wu, Zhiyong Li, Rui Xu, Yanxia Shang, Beibei Gao

**Affiliations:** a Department of Diabetic Ophthalmology, Hebei Ophthalmology Key Lab, Hebei Eye Hospital, Xingtai, Hebei, China; b Department of Ophthalmology, Peking University International Hospital, Life Science Park of Zhong Guancun, Changping District, Beijing, China.

**Keywords:** antibiotics, drug sensitivity, endophthalmitis, intravitreal injection, vitrectomy

## Abstract

Infective endophthalmitis is an ophthalmic infection that in severe cases can cause complete loss of vision. In children, the defense against infection is low and eye tissue is not fully developed, leading to increased vulnerability to endophthalmitis. Children may be unable to understand the symptoms; thus, developing a method for prevention and treatment of this disease in children is important. Therefore, we analyzed the clinical and pathogenic characteristics of infectious endophthalmitis in children and provided evidence for clinical treatment. The clinical data of 78 children (78 eyes) with infectious endophthalmitis were retrospectively analyzed. The clinical characteristics, pathogen distribution, drug sensitivity, clinical medication, and treatments were summarized and analyzed. In total, 74 (94.87%) had ocular infections caused by trauma and 75 (96.15%) were from rural townships. A total of 108 sterile specimens were examined, with a positive detection rate of 37.04%. The sensitivity rates of Gram-positive cocci and bacilli to vancomycin were 100%. The sensitivity rates of Gram-negative bacilli to ceftazidime, piperacillin/tazobactam, amikacin, gentamicin, ciprofloxacin, and levofloxacin were 100%. Of the 78 patients, 53 (67.95%) received intravitreal injection and 54 (69.23%) underwent vitrectomy. Trauma is the main factor leading to infectious endophthalmitis in children, wherein Gram-positive bacteria are the most common pathogens. Thus, a timely understanding of the pathogen and drug sensitivity is needed. Intravitreal injection and vitrectomy are effective treatments.

## 1. Introduction

Infective endophthalmitis is an ophthalmic emergency that affects vision and in severe cases can cause complete loss of vision.^[1]^ The eye tissue is not fully developed in children, and they have a lower defense against infection than adults; thus, they are relatively more vulnerable to infection.^[2]^ Once the infection progresses rapidly, it can seriously damage visual function. Failure to provide timely and effective treatment can lead to permanent loss of visual function. Additionally, children often cannot adequately understand and explain the symptoms, which can easily delay diagnosis and treatment. Therefore, the clinical diagnosis and treatment of endophthalmitis in children is more difficult.^[3]^ As a developing country with a large population base, China has a larger population of children with endophthalmitis than Europe and the United States.^[4,5]^ The clinical characteristics and bacterial spectrum of this disease may differ from those observed in other countries.^[6–8]^ However, relatively few large-sample studies on endophthalmitis in children are available. Therefore, we retrospectively analyzed and summarized case data of infectious endophthalmitis in children, including clinical characteristics, pathogen culture, drug sensitivity, drug selection, and treatment results, to provide a reference for clinical prevention and treatment.

## 2. Methodology

### 2.1. Study population

This was a non-comparative, retrospective case series. We reviewed the medical records of all patients aged < 14 years who were diagnosed with infectious endophthalmitis and treated at Hebei Provincial Eye Hospital between January 1, 2014 and December 31, 2019. This study complied with the Declaration of Helsinki and was approved by the Ethics Committee of the Hebei Provincial Eye Hospital, Hebei, China. Each patient signed an informed consent form after understanding the benefits and risks of this study, as well as alternative treatment methods.

The inclusion criteria were as follows: patients diagnosed with infectious endophthalmitis. The diagnostic criteria were pain that continued to worsen in the affected eye, decreased visual acuity, photophobia, conjunctival mixed hyperemia and edema, corneal edema, fibrous exudation or pus accumulation in the anterior chamber, vitritis, loss of red reflex, or yellow glow behind the lens. A B-ultrasound examination showing vitreous opacity was additionally considered. Extraocular signs such as lid edema, proptosis, and restricted extraocular movements were also considered^[9,10]^; with the age under 14 years old; were able to receive complete treatment and follow-up.

Exclusion criteria included cases of noninfectious endophthalmitis, such as inflammatory reaction caused by the retention of hidden lens cortex or nucleus after cataract surgery; sympathetic ophthalmia, aseptic vitreous, or pseudoendophthalmitis; uveitis with pus in the anterior chamber; toxic reaction syndrome of the anterior segment after various internal eye operations; persistent inflammatory reaction caused by the presence of concealed intrabulbar foreign body after trauma; and crystal allergic endophthalmitis.

### 2.2. Operation process and specimen culture

After the children were transferred to the hospital, primary suturing was performed under general anesthesia as soon as possible. Additionally, the vitreous or aqueous humor was aspirated and sent to the laboratory; smears were then prepared for the detection of microorganisms, followed by preparation of bacterial and fungal cultures.^[11]^ A pars plana vitrectomy was required in cases with incidences of an intraocular foreign body, traumatic retinal detachment, and displacement of the lens. At the end of the surgery, vancomycin and ceftazidime were injected into the vitreous cavity. If bacteria and fungi were not detected after culturing for 4 and 10 days, respectively, the test result was considered negative. Drug sensitivity tests were performed for patients with positive cultures.^[12]^ All surgeries were performed by the same surgeon.

### 2.3. Statistical analysis

Data were tabulated and analyzed using Microsoft Excel (Microsoft Inc., Redmond, WA, USA). Data are presented as the mean ± standard deviation. Summary and analysis of the cause and constituent ratios in patients with endophthalmitis, pathogen culture results, antimicrobial resistance, route of administration, treatment drug selection, and treatment results are presented. Survey data were evaluated as percentage calculations. Statistical analyses were performed using IBM SPSS 24 software (IBM, Armonk, NY, USA).

## 3. Results

### 3.1. Clinical characteristics

A total of 78 patients participated in this study, (age range, 1–14 years; average age, 7.04 ± 3.97 years) including 64 males (82.1%) and 14 females (17.9%), with a sex ratio of 4.6:1. Of the 78 patients, 74 (94.9%) had eye infections caused by trauma. The top 5 causes of trauma were injury caused by scissors, stationery, sticks, branches, and wires. Furthermore, 75 were from rural towns, and 3 were from urban areas (Table 1).

**Table 1:** Clinical characteristics of children with endophthalmitis.

Characteristics	Cases (n)	Proportion (%)
Sex		
Male	64	82.1
Female	14	17.9
Residential setting		
Rural	75	96.2
Urban	3	4.8
Cause		
Trauma	74	94.9
Other	4	5.1
Causes of trauma (top 10)		
Scissors	13	16.7
Stationery	9	11.5
Stick	8	10.3
Branch	5	6.4
Wire	5	6.4
Syringe	4	5.1
Nail	3	3.8
Skewer	3	3.8
Toy	2	3.6

### 3.2. Microbial culture results

A total of 108 samples were examined, 40 of which were culture-positive, with a positivity rate of 37%. In total, 39 bacteria and 1 fungus were detected in the culture-positive samples. Among the 39 bacterial strains, 23 were Gram-positive cocci, 10 were Gram-positive bacilli, and 6 were Gram-negative bacilli. Among the 23 Gram-positive cocci, 12 strains were *Streptococcus* and 9 were *Staphylococcus* (5 of which were *Staphylococcus epidermidis*) (Table 2).

**Table 2:** Distribution and proportion of pathogens isolated from samples of vitreous or aqueous humor in cases of endophthalmitis during the period 2014 to 2019.

Pathogen		Number	Percentage (%)
Gram-positive cocci		23	57.5
	Other *Streptococcus*	6	15.0
	*Staphylococcus epidermidis*	5	12.5
	*Streptococcus pneumoniae*	3	7.5
	Group A *Streptococcus*	3	7.5
	*Staphylococcus capitis*	2	5.0
	*Staphylococcus aureus*	2	5.0
	Similar Gram-positive cocci	1	2.5
	*Micrococcus luteus*	1	2.5
Gram-positive bacillus		10	25.0
	Similar Gram-positive bacilli	6	15.0
	Similar *Bacillus subtilis*	3	7.5
	Similar *Bacillus cereus*	1	2.5
Gram-negative bacillus		6	15.0
	Oligobacterium	2	5.0
	*Enterobacter cloacae*	2	5.0
	*Acinetobacter johnsonii*	1	2.5
	*Aeromonas hydrophila*/*Aeromonas caviae*	1	2.5
Fungus		1	2.5
Total		40	100

### 3.3. Drug sensitivity and resistance analysis of main pathogenic bacteria to common antibiotics

No vancomycin-resistant Gram-positive cocci were detected in this study. The 12 *Streptococcus* strains were highly sensitive (>90%) to levofloxacin, chloramphenicol, and rifampicin, moderately sensitive to penicillin (66.7%), and poorly sensitive to erythromycin, azithromycin, and clindamycin (<30%). Nine *Staphylococcus* strains were highly sensitive (100%) to amikacin and rifampicin and showed good sensitivity to chloramphenicol and levofloxacin (>75%). The sensitivities of 10 Gram-positive bacilli strains to levofloxacin and vancomycin were 100%. Six Gram-negative bacilli strains were highly sensitive (100%) to ceftazidime, piperacillin/tazobactam, amikacin, gentamicin, ciprofloxacin, and levofloxacin (Tables 3–5).

**Table 3:** Sensitivity of Gram-positive cocci to antibiotics.

Antibiotics	*Streptococcus* (n = 12)			*Staphylococcus* (n = 9)		
	Sensitive (in %)	Medium (in %)	Resistant (in %)	Sensitive (in %)	Medium (in %)	Resistant (in %)
Azithromycin	8.3	8.3	83.3	22.2	0	77.8
Erythromycin	8.3	8.3	83.3			
Clindamycin	25.0	0	75.0	44.4	11.1	44.4
Penicillin	66.7	0	33.3	11.1	0	88.9
Chloramphenicol	91.7	0	8.3	88.9	0	11.1
Rifampicin	91.7	0	8.3	100.0	0	0
Levofloxacin	100.0	0	0	77.8	22.2	0
Vancomycin	100.0	0	0	100.0	0	0
Gentamicin				55.6	22.2	22.2
Lomefloxacin				55.6	0	44.4
Cefoxitin				44.4	0	55.6
Ciprofloxacin				55.6	0	44.4
Tobramycin				77.8	0	22.2
Amikacin				100.0	0	0

**Table 4:** Sensitivity of Gram-positive bacilli to antibiotics.

Antibiotics	Gram-positive bacilli (n = 10)		
	Sensitive (in %)	Medium (in %)	Resistant (in %)
Penicillin	0	0	100.0
Ceftazidime	20.0	0	80.0
Cefazolin	30.0	0	70.0
Cefotaxime	40.0	20.0	40.0
Cefuroxime	40.0	0	60.0
Rifampicin	40.0	0	60.0
Cefoperazone	50.0	30.0	20.0
Clindamycin	60.0	0	40.0
Amikacin	70.0	0	30.0
Azithromycin	70.0	0	30.0
Ciprofloxacin	80.0	0	20.0
Tobramycin	80.0	0	20.0
Chloramphenicol	80.0	20.0	0
Cefoxitin	80.0	0	20.0
Gentamicin	80.0	0	20.0
Lomefloxacin	100.0	0	0
Levofloxacin	100.0	0	0
Vancomycin	100.0	0	0

**Table 5:** Sensitivity of Gram-negative bacilli to antibiotics.

Antibiotics	Gram-negative bacilli (n = 6)		
	Sensitive (in %)	Medium (in %)	Resistant (in %)
Cefoperazone	50.0	16.7	33.3
Cefotaxime	50.0	50.0	0
Amtreonam	50.0	0	50.0
Imipenem	83.3	0	16.7
Chloramphenicol	83.3	16.7	0
Amikacin	100.0	0	0
Ceftazidime	100.0	0	0
Ciprofloxacin	100.0	0	0
Gentamicin	100.0	0	0
Levofloxacin	100.0	0	0
Piperacillin/Tazobactam	100.0	0	0

### 3.4. Drug selection and administration route

The main drug treatments for the 78 cases of infectious endophthalmitis were systemic administration (including intravenous and oral administration), local application of eye drops or ointments, and local injection (including intravitreal, subconjunctival, and retrobulbar injections). Among them, 77 patients (98.7%) received systemic administration, 78 (100%) received local eye drops, and 59 (75.6%) received local injections. A total of 58 patients (74.4%) received a combination of the 3 drug delivery routes. The top 5 systemic medications used were cefepime (37 cases, 48.1%), cefuroxime (34, 44.2%), cefazolin (15, 19.5%), cefotaxime (5, 6.5%), and clindamycin (4, 5.2%). The 3 antibiotics used for topical anterior drip were levofloxacin (71, 91.0%), tobramycin (60, 76.9%), and rifampicin (27, 34.6%). The top 3 local injections included vancomycin (58, 98.4%), ceftazidime (16, 27.1%), and tobramycin (5, 0.8%). All cases were adjusted based on the drug sensitivity results, and the coincidence rate between drug selection and sensitivity was 100%.

### 3.5. Treatment results

Of the 78 patients in this study, 1 patient with mild infective endophthalmitis was treated with systemic medication only, resulting in considerable improvement in vision and discharge, and 53 patients received intravitreal drug injections. Postoperative observations revealed that 21 patients showed improved symptoms, whereas 32 did not. Finally, 22 patients underwent vitrectomy immediately after admission, and 2 patients underwent intraocular volume removal owing to uncontrolled inflammation.

## 4. Discussion

Infectious endophthalmitis is a serious ophthalmic emergency that affects visual function. Inflammatory cell infiltration induced by bacterial infection and the action of bacterial toxins severely damage the structure and function of the retina.^[13]^ Infectious endophthalmitis in children is more complex than that in adults and has unique characteristics.^[7]^ First, the ocular tissue structure in children is fragile, and the ability of children to resist trauma is poor. They react violently to various injuries. Endophthalmitis can develop easily after ocular perforation. Severe inflammatory reactions damage not only the retina but also the ciliary body, thereby decreasing aqueous humor secretion, which results in low intraocular pressure and atrophy of the eyeball. Endophthalmitis also has an acute onset and rapid progression. If not treated promptly or appropriately, bacteria and their toxins spread throughout the body, leading to systemic infection. Finally, due to the poor self-protection ability of children. Their reactions to various injuries can be more extreme than that of adults. Oftentimes, children do not cooperate well and cannot accurately describe their medical history, which can easily lead to delays in diagnosis and treatment that seriously affect the recovery of visual function.

The results of the present study showed that traumatic endophthalmitis accounted for 94.9% of endophthalmitis cases and was the primary cause of endophthalmitis in children. After ocular trauma, the integrity of the ocular wall can be compromised, allowing pathogenic bacteria to enter the inner eye and cause infection. Most children presenting with eye trauma are male, which may be related to their hyperactivity and extensive physical activity.^[14]^ Our study found that the main cause of eye injury was consistent with that reported by Wu et al^[3]^ who reported that scissors are the most common cause of eye injury. Parents should monitor their children and improve their capability to identify dangerous objects as well as reduce their exposure to such objects. Of the 78 cases of endophthalmitis, 75 were from rural villages and towns, whereas 3 were from urban areas. This may be attributed to different factors: majority of patients reside in rural areas, a lack of adult supervision, and a poor awareness of safety education. Therefore, it is crucial to enhance injury-prevention awareness, strengthen safety education, and increase awareness of eye trauma hazards.

Our study found that the detection rate of Gram-positive bacteria, which were the main pathogenic bacteria causing infectious endophthalmitis in children, was 59%. Streptococci represented the majority of the Gram-positive isolates, which is consistent with findings of the BHAGAT study,^[6]^ followed by *S epidermidis. Staphylococcus epidermidis* is widely distributed in nature, a member of the normal flora, and an opportunistic pathogen in the conjunctival sac, skin, and other parts of the body. Changes in the immune status or ocular surface microenvironment, as well as eye trauma, can facilitate the entry of streptococci into the eye through damaged areas and represent important factors in eye infections.^[15]^

According to the statistics of drug sensitivity tests conducted in our hospital over 5 years, there was no evidence of resistance of Gram-positive cocci to vancomycin. Vancomycin can be selected to treat infections with bacteria that have a vancomycin sensitivity rate of 100% when considering drug-resistant bacterial infections or other drug failures. The sensitivity rates of *Staphylococcus* and *Streptococcus* to rifampicin were 100% and 91.7%, respectively, and that of *Staphylococcus* to levofloxacin was 100%. Therefore, rifampicin and levofloxacin can be used as the initial empirical drugs of choice for treating infections with Gram-positive cocci. Gram-negative bacilli were highly sensitive to amikacin, gentamicin, ciprofloxacin, levofloxacin, ceftazidime, and piperacillin/tazobactam. These drugs can be used as empirical choices for treating infections with Gram-negative bacilli.

Rational use of antibacterial drugs plays an important role in the treatment of infectious endophthalmitis. Regarding the use of drug treatment against endophthalmitis, in addition to administering early treatment, it should be used in combination. Owing to the blood–eye barrier, local and systemic medications can be administered simultaneously. In this study, 58 patients (74.4%) were treated with systemic administration, local topical administration, and local injections (including intravitreal, subconjunctival, and retrobulbar injections) of antibacterials. Regarding drug selection, the main systemic drugs used were cefotaxime (48.1%) and cefuroxime (44.2%), considering that macrolide antibiotics cannot be used for systemic administration in children. Both drugs are broad-spectrum antibacterial drugs targeting common bacteria. The most common topical eye drop used contained levofloxacin (91%). In terms of the ocular permeability of drugs, levofloxacin administration can achieve effective drug concentrations in the aqueous humor after the use of topical eye drops, with ideal intraocular penetration. According to the drug sensitivity results reported by our hospital, the sensitivity rate of *Staphylococcus* and Gram-positive and Gram-negative bacilli to levofloxacin was 100%, and the sensitivity rate of *Streptococcus* to levofloxacin was 77.8% (intermediate, 22.2%), indicating high sensitivity. Therefore, levofloxacin can be considered as one of the first-choice drugs for empirical treatment in the early stages of infectious endophthalmitis. However, considering the toxic effects of quinolones on bone development in children, their use is prohibited in adolescents younger than 18 years of age. Therefore, levofloxacin was only used topically. Intravitreal injection of drugs represents an important treatment option for endophthalmitis. The intravitreal injection of vancomycin can facilitate direct entry of drugs into the vitreous cavity, ensuring an effective therapeutic concentration in the vitreous body and aqueous humor. Several studies have shown that timely intravitreal injections of antibiotics can effectively treat traumatic infectious endophthalmitis in children.^[16,17]^ Of the 78 patients in the present study, 59 received an intravitreal injection of vancomycin (98.4%) or a combination of vancomycin and ceftazidime (98.4%). Intravitreal injection of vancomycin can rapidly achieve effective therapeutic drug concentrations in the eye. Gram-positive bacteria are sensitive to vancomycin, whereas Gram-negative bacteria are not. Ceftazidime has good antibacterial activity against Gram-negative bacteria. Therefore, in the early stages of the disease, a combination of broad-spectrum antibiotics can be selected to address the possibility of infection with Gram-positive and Gram-negative bacteria to better control endophthalmitis development. Peng et al^[18]^ retrospectively analyzed 27 cases of endophthalmitis treated with vancomycin and found that the detection rates of Gram-positive and Gram-negative bacteria are 67% and 33%, respectively. Therefore, using vancomycin intravitreal injection or vitrectomy alone can cause uncontrollable endophthalmitis, and broad-spectrum combination treatment is recommended in the early stages of the disease.

In this study, intravitreal injection therapy was initially administered to treat early and mild infectious endophthalmitis, and the condition of the patient was closely monitored after surgery. If inflammation cannot be relieved and gradually worsens, a timely vitrectomy should be performed. Of the 78 patients in this study, 54 underwent vitrectomy, suggesting that the treatment of endophthalmitis should be combined with surgery, in addition to the timely administration of antibacterial drugs.

Currently, only a few studies have evaluated endophthalmitis in children. Our study comprehensively described the clinical characteristics, pathogen distribution, drug sensitivity results, clinical medications, and treatment measures for endophthalmitis in children, providing sufficient evidence for its clinical treatment.

In summary, effective preventive measures for eye injuries in children in rural areas, especially boys, are key to preventing traumatic endophthalmitis. Society must consider this issue, improve safety awareness, strengthen the popularization and promotion of preventive measures for eye injuries, and perform targeted preventive measures to reduce the incidence of eye injuries. We conducted etiological observations in children with bacterial endophthalmitis to help clinicians better understand the etiological background and provide evidence-based targeted treatment in clinical settings before bacterial culture and drug sensitivity results are issued. According to our statistical results, no single antibacterial agent is currently effective against all pathogenic bacteria; therefore, combination medication should be considered before determining the pathogen and its drug sensitivity. Intravitreal drug injections and vitrectomy remain the best options for treating infectious endophthalmitis in children, enabling such patients to receive more reasonable treatment and maximize recovery of visual function.

Our study has some limitations. It is a retrospective design and there is no control group. In the future, we will design prospective case–control studies based on this one to observe the efficacy of various treatment methods for infectious endophthalmitis in children.

## Author contributions

**Formal analysis:** Peipei Jia, Jiayin Qin, Yixiang Wu, Zhiyong Li, Rui Xu, Yanxia Shang, Beibei Gao.

**Investigation:** Peipei Jia, Jiayin Qin, Yixiang Wu, Zhiyong Li, Rui Xu, Yanxia Shang, Beibei Gao.

**Writing – original draft:** Peipei Jia, Jiayin Qin.

**Conceptualization:** Xiaolu Cao.
